# Salivary microbial dysbiosis is associated with systemic immune factors in oral squamous cell carcinoma patients

**DOI:** 10.1097/MD.0000000000044217

**Published:** 2025-09-12

**Authors:** Jiazhi Xu, Dongxiao Nong, Xiaolin Nong, Jun Zhao, Rui Bai, Chan Tang, Jiayi Hang

**Affiliations:** a Department of Oral and Maxillofacial Surgery, College of Stomatology, Hospital of Stomatology, Guangxi Medical University, Nanning, Guangxi Province, China; b Department of Otorhinolaryngology-Head and Neck Surgery, First Affiliated Hospital of Guangxi Medical University, Guangxi Medical University, Nanning, Guangxi Province, China; c Guangxi Key Laboratory of Oral and Maxillofacial Rehabilitation and Reconstruction, Nanning, Guangxi Province, China.

**Keywords:** cytokines (IL-2, IL-10, IFN-γ), immune response, microbial dysbiosis, oral squamous cell carcinoma, salivary microbiome

## Abstract

This study investigates the alterations in the composition of the oral microbiome in individuals with oral squamous cell carcinoma (OSCC) and examine the relationship between the oral microbiome and immune-related variables in the tumor immunological microenvironment of the host. The expression levels of immune factors interleukin-2 (IL-2), interleukin-10 (IL-10), and interferon-γ (IFN-γ) in the peripheral blood of OSCC patients and healthy volunteers were investigated using the enzyme-linked immunosorbent test (ELISA). Saliva samples were obtained from both OSCC patients and healthy control participants. The bacterial 16SrRNA gene was then analyzed using high-throughput sequencing to compare the composition and quantity of oral flora between OSCC patients and healthy volunteers. The bacteria that exhibited notable differences were compared with each other. Analyzed the correlation between the systemic immunological state of the host. The serum levels of IL-2 and IFN-γ were significantly greater in the OSCC group compared to the healthy controls (HC) group (IL-2, *P*<.001, IFN-γ, *P*<.01). In contrast, the serum IL-10 expression level in the OSCC group was significantly lower than that in the HC group (*P* <.01). The bacterial genera that differ between the OSCC group and the HC group are associated with the host immunological markers. Peptostreptococcus exhibited a noteworthy positive correlation with IL-2, but Thermus demonstrated a negative correlation with IL-2. The presence of IL-10 was shown to be positively associated with the genera Oralobacter and negatively associated with the genus Eikenella. The genus Peptostreptococcus had a notable positive connection with IFN-γ and a negative correlation with unclassified Actinobacteria. Significant differences in immune factor expression and oral microbiota were found between OSCC patients and HC. Microbial dysbiosis, particularly involving Fusobacterium and Veillonella, may influence OSCC progression by modulating the immune microenvironment.

## 1. Introduction

Oral squamous cell carcinoma (OSCC) is a prevalent form of cancer in humans.^[1]^ OSCC is unique compared to other sections of the body due to its occurrence in the intricate anatomical position of the maxillofacial region.^[2]^ Furthermore, due to the proximity of the disease site to vital tissues and organs such as the base of the skull and the throat, and the abundance of nerves and blood vessels in this area compared to other parts, several factors contribute to the elevated recurrence and metastasis rates, as well as the high mortality and unfavorable prognosis.^[3,4]^ Despite significant advancements in therapeutic methods including radiotherapy and chemotherapy, the 5-year survival rate remains discouraging, with a rate of <50%.^[5,6]^ Gaining a more comprehensive comprehension of the mechanisms behind the occurrence and progression of OSCC, as well as the pursuit of more precise and efficient cancer diagnostic markers, may facilitate the development of novel therapeutic alternatives.

Cytokines exhibit a wide array of biological roles. The majority of cytokines consist of monomers, however a small number have a dimer or trimer structure. They have the ability to exert their actions through autocrine, paracrine, and endocrine mechanisms, demonstrating pleiotropic and overlapping effects.^[7]^ In living organisms, cytokines interact in a complex regulatory network that can generate antagonistic effects among themselves.^[8]^ IL-2, often referred to as interleukin-2 or T cell growth factor, is mainly synthesized by T lymphocytes and exerts its biological effects through autocrine and paracrine mechanisms.^[9,10]^. The primary job of this substance is to initiate the activation of T cells, stimulate the production of cytokines, boost the proliferation of NK cells, enhance the killing ability of NK cells, and induce the formation of LAK cells. Its major function is to support cellular immunity.^[11]^ Moreover, it can enhance the growth of B cells and the production of antibodies, while also stimulating macrophages to support humoral immunity. Over the past 3 decades, it has been established that IL-2 has demonstrated significant effectiveness in immunotherapy for combating tumors and infections.^[12]^ IL-10 is primarily produced by T2 cells and mononuclear macrophages.^[13]^ It mainly prevents the synthesis of pro-inflammatory cytokines and the development of MHCI and B7 molecules. Additionally, it hinders T cells from producing IL-2, IFN-γ, and other cytokines.^[14,15]^ IL-10 is a crucial cytokine with anti-inflammatory properties that might hinder the functioning of different immune cells. The primary physiological importance of IL-10 is its ability to inhibit both specific and nonspecific immune responses, hence minimizing tissue damage. Additionally, IL-10 plays a role in promoting immunological tolerance.^[16]^

The human oral microbiota has a vital function in controlling host energy metabolism, immunity, inflammation, and various other characteristics. The human body has a microecosystem that is of utmost importance.^[17]^ The mouth cavity, being the first part of the digestive tract, contains many places where germs can attach and grow, making it a crucial environment for microbes in the human body.^[18,19]^ The oral flora is a very complex microbial community within the human body that interacts intricately with the immune and inflammatory systems.^[20]^ A stable microbiome maintains a balanced and constantly changing relationship with the host, which is essential for human health.^[21]^ An ecological imbalance occurs when this equilibrium system becomes abnormal.^[22]^ There are multiple reasons that can cause an imbalance in microbiota, and both internal and external stressors can result in ongoing changes in the microflora.^[23]^ Hence, achieving a thorough comprehension of the intricate microbial–host interactions and the factors that cause human morbidity continues to be a difficult task.

The primary objective of this study is to conduct an initial examination of immune expression in the peripheral blood of patients with OSCC by assessing the levels of immune-related molecules such as IL-2, IL-10, and IFN-γ. In addition, we collected and analyzed oral saliva samples from patients with OSCC and healthy individuals utilizing amplicon sequencing of the 16S rRNA gene. The statistical analysis was conducted to examine the link between host immune-related parameters and OSCC development and early detection, in order to provide fresh guidelines and experimental evidence.

## 2. Materials and methods

### 2.1. Patient and specimens

The specimens used in this investigation were obtained from the maxillofacial surgery ward of the Affiliated Stomatological Hospital of Guangxi Medical University. The patients included in the study were admitted between August 2022 and September 2023. All subjects received unambiguous pathology results and a definitive diagnosis of squamous cell carcinoma. The Medical Ethics Committee of the Affiliated Stomatology Hospital of Guangxi Medical University authorized all samples taken in this investigation. The ethics number assigned to this study was Audit 2022067. Prior to the surgical procedure, it is essential to engage in communication with the individual involved, and thereafter obtain the patient’s signature on the consent form.

The patients who were registered satisfied the following criteria: the pathological diagnostic indicated the presence of OSCC, which includes squamous cell carcinoma in the tongue, cheek, mouth, and gum. Fill out comprehensive treatment records and medical history; thorough and comprehensive clinicopathological data, including pathology number, site of OSCC, tumor-node-metastasis stage, and other relevant information. Provide comprehensive personal details, including address, telephone number, etc, for further communication; The systemic state is capable of enduring the related sampling and treatment approaches. All participants willingly provided their signature on the informed consent form, which received approval from the Ethics Department of Stomatology Hospital of Guangxi Medical University.

The criteria for including individuals in the healthy control group: Patients in the healthy control group should exhibit a state of oral health that is free from gingival redness, ulcers, and other oral disorders. Absence of obesity, diabetes, cardiovascular diseases, metabolic diseases, and other systemic ailments; age: mentally well, without any psychiatric disorders; Absence of contagious illnesses; Provide comprehensive personal details, including address, telephone number, etc, for further communication. The systemic state is capable of enduring the related sampling and treatment approaches. Each subject willingly provided informed consent.

### 2.2. Enzyme-linked immunosorbent assay

Prior to the patient’s surgery, blood samples were collected and subjected to centrifugation with a force of 1000 g at a temperature of 4°C for a duration of 15 minutes. Subsequently, the samples were stored at a temperature of −80°C for subsequent tests. Equal quantities of serum were used to assess the levels of interleukin-2 (IL-2), interleukin-10 (IL-10), and interferon-γ (IFN-γ). The liankebio (Hangzhou, China) ELISA kit was utilized for testing, and the experimental procedures were meticulously conducted in line with the instructions provided with the kit.

### 2.3. Saliva sample collection and bacterial DNA extraction

Participants were instructed to refrain from food and alcohol consumption for at least 30 minutes prior to saliva collection. Unstimulated saliva samples were then collected from each participant and immediately transferred into sterile centrifuge tubes containing 70% ethanol for preservation. The samples were promptly stored at −80°C until further DNA extraction and sequencing procedures.

### 2.4. Amplicon sequencing analysis of bacterial 16S rRNA gene

The PCR reaction system was set up using 30 ng of validated genomic DNA samples and the associated fusion primers. The PCR reaction parameters were established for the purpose of PCR amplification. The PCR amplification products were purified using Agencourt AMPure XP magnetic beads, dissolved in Elution Buffer, and labeled. The database has been finalized. The library’s fragment range and concentration were assessed using the Agilent 2100 Bioanalyzer. The libraries that met the necessary requirements were subjected to sequencing utilizing sequencers, based on the size of the fragment that was inserted. Data filtering for disembarkation, ensuring high-quality results. Data was cleaned in preparation for subsequent analysis. Reads were split into tags based on the overlap between them. Cluster tags were grouped into operational taxonomic units (OTUs) and compared with a database to annotate species. Using the OTU and annotation results, a study of sample species complexity, inter-group species differences, association analysis, and model prediction were conducted.

### 2.5. Analysis of oral flora diversity

The dilution curve was constructed by plotting the relative proportion of each sample’s OTU obtained from the sequence results. Specifically, the expected value of each Alpha index was calculated when a certain number of tags (n) were extracted (where n is less than the total number of measured read sequences). The curve was then plotted based on the expected value of the Alpha index corresponding to a group of n values (typically an arithmetic series that is smaller than the total number of sequences). The initial data were categorized into phylum and genus levels in order to examine the proportional representation of oral microorganisms in the OSCC group and healthy controls (HC) group, and to assess their correlation with immune-related parameters.

### 2.6. Statistical methods

Data analysis was conducted using the statistical software SPSS 21 (Chicago), and statistical visualizations were generated using GraphPad Prism 9. The outcomes for the majority of categorical variables are shown as counts (percentages), whereas the outcomes for continuous variables are presented as the mean plus or minus the standard deviation. The Chi-square test was employed to assess the relative disparities between the category variables. The unpaired samples were analyzed using either the Student *t*-test or the Mann–Whitney *U* test to assess the differences in the continuous variables across the independent samples. Additionally, adjustments were made for the rate of false discovery (FDR). The Spearman correlation coefficient test was employed to conduct the correlation study. All significance tests were 2-tailed, and a *P*-value <.05 was deemed statistically significant.

## 3. Results

### 3.1. Physiological characteristics of study subjects

In this study, 50 clinical subjects were selected, among which 36 were males, accounting for 72% of the total. There were 14 female patients, accounting for 28% of the total number, and the clinical trial group was mainly male. Tumor-node-metastasis stages were classified according to the UICC (8th edition) standard. Among the OSCC patients, 10 cases (20%) were classified as stage I, 18 cases (36%) as stage II, 10 cases (20%) as stage III, and 12 cases (24%) as stage IV. According to OSCC differentiation degree criteria, 10 cases (20%) were poorly differentiated, 15 cases (30%) were moderately differentiated, and 25 cases (50%) were highly differentiated. Most of the patients in OSCC test group were highly differentiated. According to preoperative examination and postoperative pathological examination, 21 cases in OSCC test group had lymphatic metastasis, accounting for 42% of the total, and 29 cases without lymphatic metastasis, accounting for 58% of the total. In the control group, 31 males accounted for 62% of the total, and 19 females accounted for 38% of the total. The healthy control group was 48.04 ± 11.84 years old. The detailed characteristics of the cohort are summarized in Table 1.

**Table 1:** Summary of clinical data of patients with oral squamous cell carcinoma and healthy controls.

Parameter	OSCC (n = 50)	HC (n = 50)	*P*-value
Age (years; mean ± SD)	54.90 ± 13.02	48.04 ± 11.84	.897
Gender (n, %)			
Male	36 (72%)	31 (62%)	–
Female	14 (28%)	19 (38%)	.288
TNM (n, %)			
Stage I	10 (20%)	–	–
Stage II	18 (36%)	–	–
Stage III	10 (20%)	–	–
Stage IV	12 (24%)	–	–
Pathology (n, %)			
Poor	10 (20%)	–	–
Moderate	15 (30%)	–	–
Well	25 (50%)	–	–
Lymphatic metastasis (n, %)			
No	29 (58%)	–	–
Yes	21 (42%)	–	–

HC = healthy controls, OSCC = oral squamous cell carcinoma, SD = standard deviation, TNM = tumor-node-metastasis.

### 3.2. IL-2, IL-10, and IFN-γ were differentially expressed in peripheral blood

**Figure 1.**
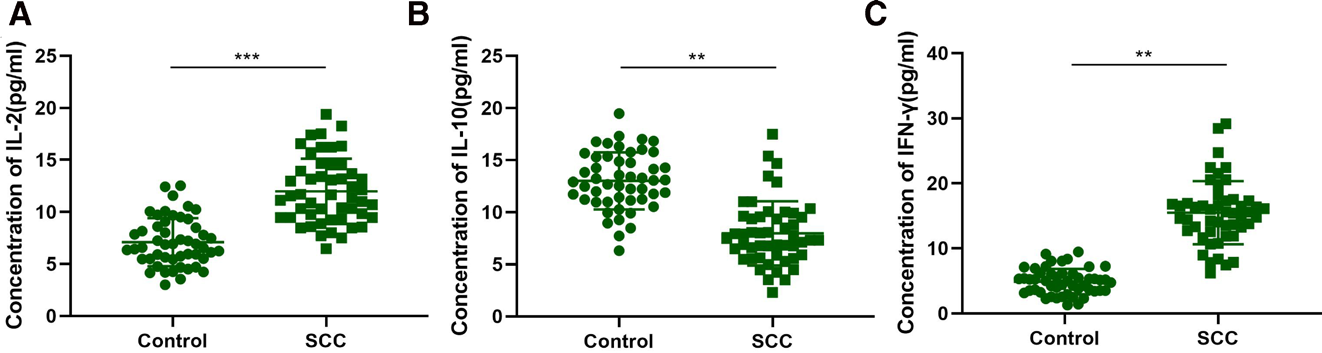
Differential expression of immune factors IL-2, IL-10, and IFN-γ in the serum of OSCC patients and healthy controls. (A) The concentration of IL-2 was significantly higher in the serum of OSCC patients compared to healthy controls (***P <.001). (B) IL-10 levels were significantly lower in OSCC patients compared to healthy controls (**P <.01). (C) IFN-γ concentrations were significantly elevated in OSCC patients compared to healthy controls (**P <.01). IFN-γ = interferon-γ, IL-2 = interleukin-2, IL-10 = interleukin-10, OSCC = oral squamous cell carcinoma.

In this part of the study, the expression of immunorelated factors IL-2, IL-10 and IFN-γ in serum was detected. As seen in Table 2, The expression of IL-2 in peripheral blood of OSCC group and HC group was 11.98 ± 3.13 pg/mL and 7.10 ± 2.31 pg/mL, respectively. The difference between the 2 groups was statistically significant (*P* <.001). The expression levels of IL-10 in serum of OSCC group and HC group were statistically different (*P* <.01), which were 7.97 ± 3.08 pg/ml and 13.01 ± 2.74 pg/mL, respectively. Serum IFN-γ expression levels in OSCC group and HC group were 14.45 ± 4.78 pg/mL and 4.88 ± 1.97 pg/mL, respectively, and the difference between the 2 groups was statistically significant (*P* <.01) (Fig. 1).

**Table 2:** Expression of immune factors IL-2, IL-10 and IFN-γ in OSCC group and HC group.

Biomarker	OSCC (pg/ml), (mean ± SD)	HC (pg/ml), (mean ± SD)	*P*-value
IL-2	11.98 ± 3.13	7.10 ± 2.31	<.001
IL-10	7.97 ± 3.08	13.01 ± 2.74	<.01
IFN-γ	14.45 ± 4.78	4.88 ± 1.97	<.01

HC = healthy controls, IFN-γ = interferon-γ, IL-2 = interleukin-2, IL-10 = interleukin-10, OSCC = oral squamous cell carcinoma, SD = standard deviation.

### 3.3. The expression of IL-2, IL-10, and IFN-γ were correlated with the clinicopathological features of OSCC

**Figure 2.**
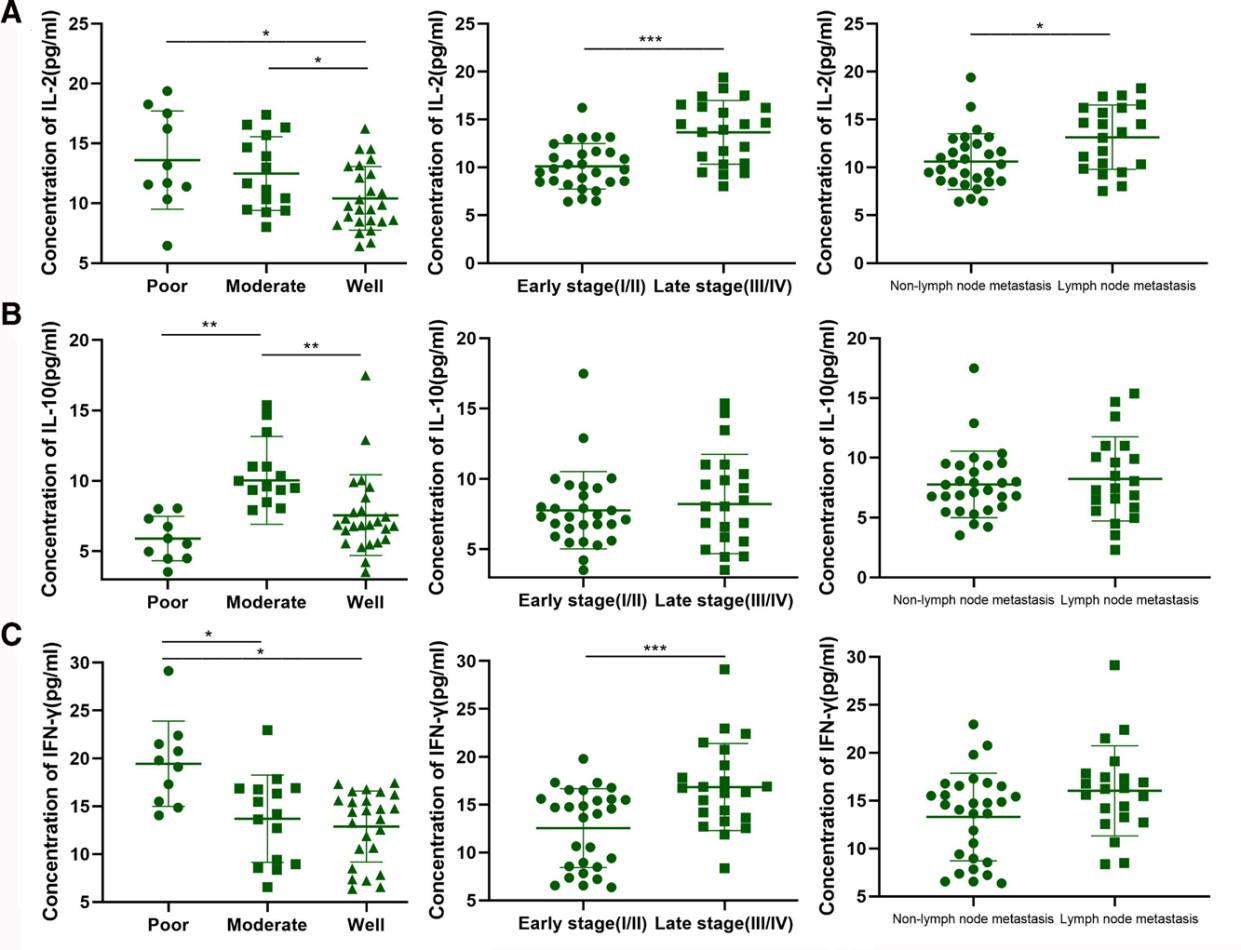
Correlation of serum IL-2, IL-10, and IFN-γ levels with clinicopathological features of OSCC patients. (A) The concentration of IL-2 in OSCC patients’ serum based on differentiation (left), clinical stage (middle), and lymph node metastasis status (right). IL-2 levels were significantly higher in moderately differentiated and late-stage tumors compared to well-differentiated and early-stage tumors (*P <.05, ***P <.001). IL-2 expression was also significantly elevated in patients with lymph node metastasis compared to those without (*P <.05). (B) Serum IL-10 levels were significantly higher in patients with poor differentiation compared to those with moderate and well differentiation (**P <.01). However, no significant differences in IL-10 levels were observed between clinical stages or lymph node metastasis groups. (C) IFN-γ levels were significantly higher in poorly and moderately differentiated tumors compared to well-differentiated tumors (*P <.05), and in late-stage patients compared to early-stage patients (***P <.001). There was no significant difference in IFN-γ levels between lymph node metastasis and nonmetastasis groups. IFN-γ = interferon-γ, IL-2 = interleukin-2, IL-10 = interleukin-10, OSCC = oral squamous cell carcinoma.

As shown in Table 3, serum IL-2 expression in OSCC had statistical significance between differentiation groups and lymphatic metastasis groups (*P* <.05), and also had statistical difference between staging groups (*P* <.001). The expression level of IL-10 in OSCC serum was statistically significant between the differentiated groups (*P *<.001), but not between the stage groups and lymphatic metastasis groups. The expression level of IFN-γ in OSCC serum was significantly different among differentiated groups (*P *<.05), and significantly different among staging groups (*P* <.001), but not among lymphatic metastasis groups (Fig. 2).

**Table 3:** Expression of immune factors IL-2, IL-10 and IFN-γ in different clinical feature groups in OSCC group.

Parameter		IL-2 (pg/mL)			IL-10 (pg/mL)			IFN-γ (pg/mL)		
		n	Mean ± SD	*P*	n	Mean ± SD	*P*	n	Mean ± SD	*P*
Pathology	Poor	10	13.60 ± 4.11	.025	10	5.90 ± 1.58	.004	10	19.45 ± 4.46	.005
	Moderate	15	12.47 ± 3.09		15	10.03 ± 3.11		15	13.70 ± 4.56	
	Well	25	10.40 ± 2.66		25	7.56 ± 2.87		25	12.89 ± 3.70	
TNM	I/II	28	10.09 ± 2.38	<.001	28	7.77 ± 2.74	.570	28	12.55 ± 4.11	<.001
	III/IV	22	13.65 ± 3.33		22	8.22 ± 3.53		22	16.85 ± 4.55	
Lymphatic metastasis	No	29	10.59 ± 2.91	.044	29	7.77 ± 2.78	.217	29	13.30 ± 4.57	.315
	Yes	21	13.14 ± 3.36		21	8.24 ± 3.51		21	16.03 ± 4.71	

HC = healthy controls, IFN-γ = interferon-γ, IL-2 = interleukin-2, IL-10 = interleukin-10, OSCC = oral squamous cell carcinoma, SD = standard deviation, TNM = tumor-node-metastasis.

### 3.4. Analysis of oral flora diversity

**Figure 3.**
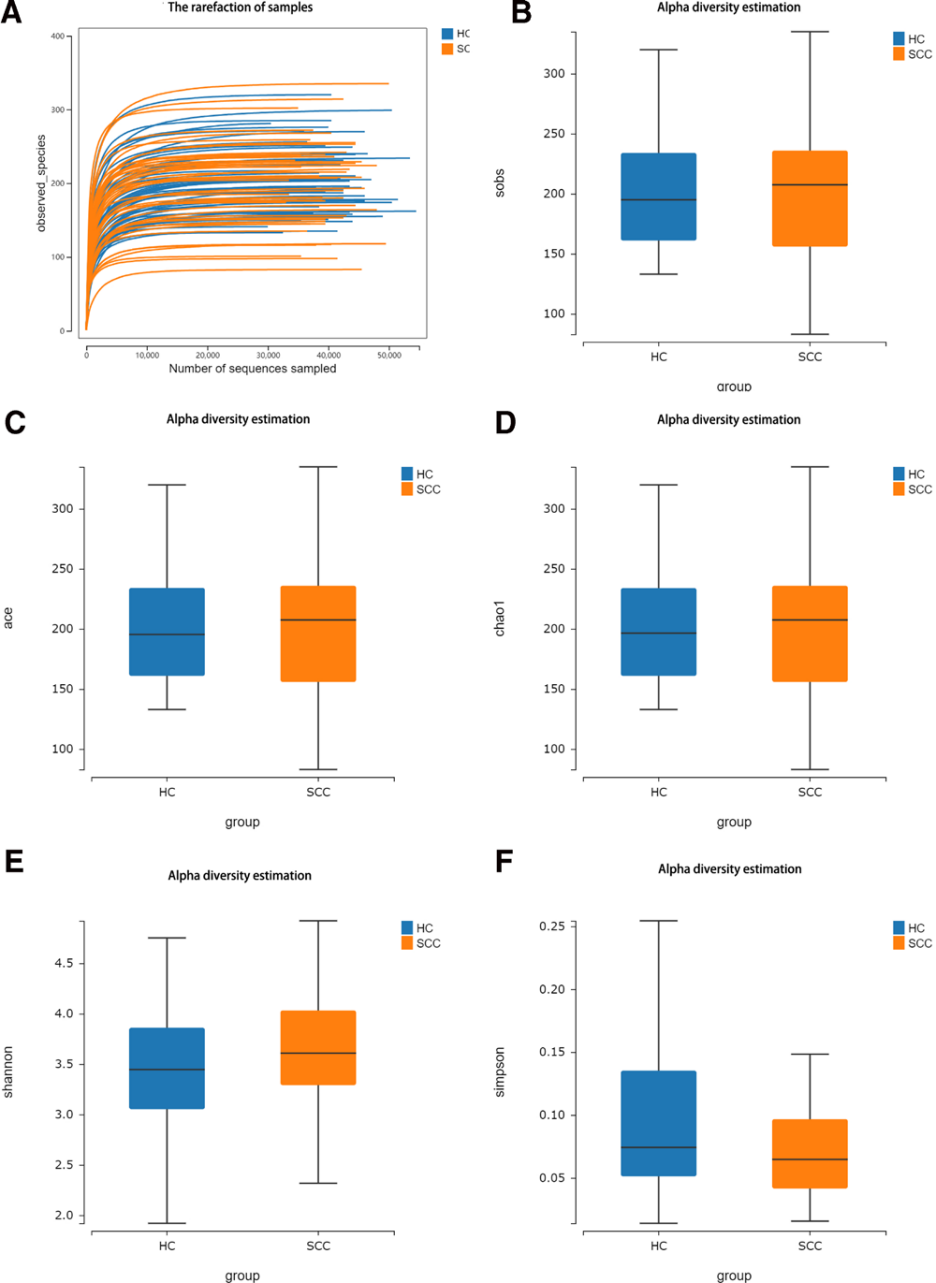
Alpha diversity analysis of the oral microbiome in OSCC patients and healthy controls. (A) Rarefaction curves showing the number of observed species (OTUs) at different sequencing depths in OSCC patients (SCC) and HC. (B) Sobs index, representing observed species richness, comparing the OSCC and HC groups. (C) ACE index, which estimates species richness, showing comparisons between the OSCC and HC groups. (D) Chao1 index, another estimator of species richness, comparing OSCC patients and healthy controls. (E) Shannon diversity index, indicating the overall diversity of microbial communities in OSCC patients versus healthy controls. (F) Simpson index, representing the evenness and diversity of the microbial communities, comparing the OSCC and HC groups. ACE = abundance-based coverage estimator, HC = healthy controls, OSCC = oral squamous cell carcinoma, OTUs = operational taxonomic units, SCC = squamous cell carcinoma.

The oral flora of OSCC patients and HC oral flora samples were analyzed. After obtaining the initial Effective Tags, that is, valid data, Uparse software was used for cluster analysis. In this experiment, the obtained sequences were clustered into OTUs with 97% consistency. The average Tag quantity measured by the 2 groups of samples and the OUT quantity obtained after cluster analysis are shown in Table 1. Sobs, abundance-based coverage estimator and Chao1 indexes are used to analyze the relative abundance of microbial communities, while Shannon index and Simpson diversity index are used to analyze the diversity of microbial communities, as shown in Table 4 and Figure 3.

**Table 4:** Oral microflora sequencing results of oral squamous cell carcinoma group and healthy control group.

Samplename	Tag_num	Otu_num	Sobs	Chao1	Ace	Shannon	Simpson	Coverage
OSCC	41,825.860	202.420	202.420	202.515	202.564	3.392	0.119	0.99999
HC	40,972.400	199.420	199.420	199.555	199.567	3.597	0.094	0.99998

HC = healthy controls, OSCC = oral squamous cell carcinoma.

### 3.5. Difference analysis of oral flora between OSCC group and HC group

**Figure 4.**
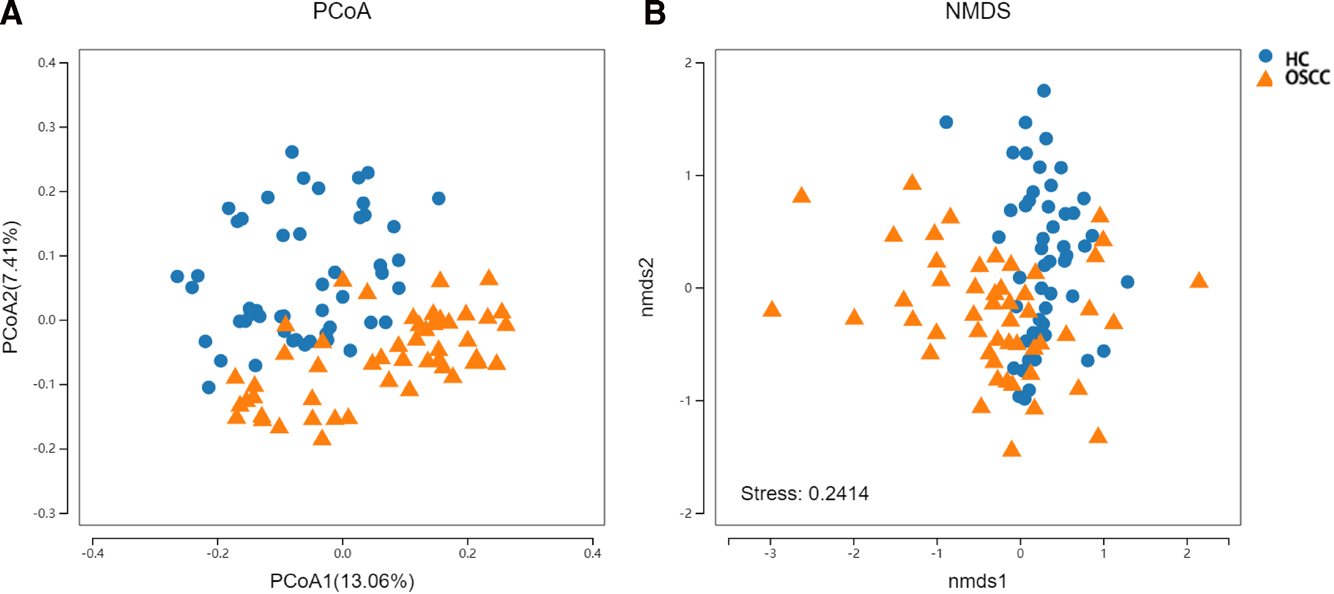
Beta diversity analysis of oral microbial communities between OSCC patients and healthy controls. (A) PCoA plot based on the Unweighted UniFrac distance matrix. The proportions of variation explained by PCoA1 and PCoA2 are 13.06% and 7.41%, respectively. The microbial composition of the OSCC group (orange triangles) and the HC group (blue circles) shows significant separation, indicating distinct microbial profiles between the groups (*P* <.001). (B) NMDS analysis showing clustering of OSCC and HC groups. The stress value of 0.2414 indicates a good fit for the model, with the microbial composition in OSCC patients differing significantly from that of healthy controls (*P* <.001). HC = healthy controls, NMDS = nonmetric multidimensional scaling, OSCC = oral squamous cell carcinoma, PCoA = principal coordinate analysis.

When analyzed in the UniFrac distance, or Unweighted UniFrac matrix, the proportions of the data in the PCoA1 dimension and PCoA2 dimension were 13.06% and 7.41% respectively, and the difference between the 2 groups was statistically significant (*P* <.001). PCoA analysis showed that OSCC group and HC group had statistically significant differences in flora species. At this time, the data will be analyzed by nonmetric multidimensional scaling method. The samples of multidimensional space will be simplified to low-dimensional space for positioning, analysis and classification, which can be used to test whether the differences between groups are significantly greater than the differences within groups, so as to judge whether the groups are meaningful. The results showed that the difference of flora structure between OSCC group and HC group was greater than the difference of flora structure within the group, and the grouping was statistically significant (*P* <.001) (Fig. 4).

### 3.6. Analysis of relative abundance of oral flora in OSCC group and HC group

**Figure 5.**
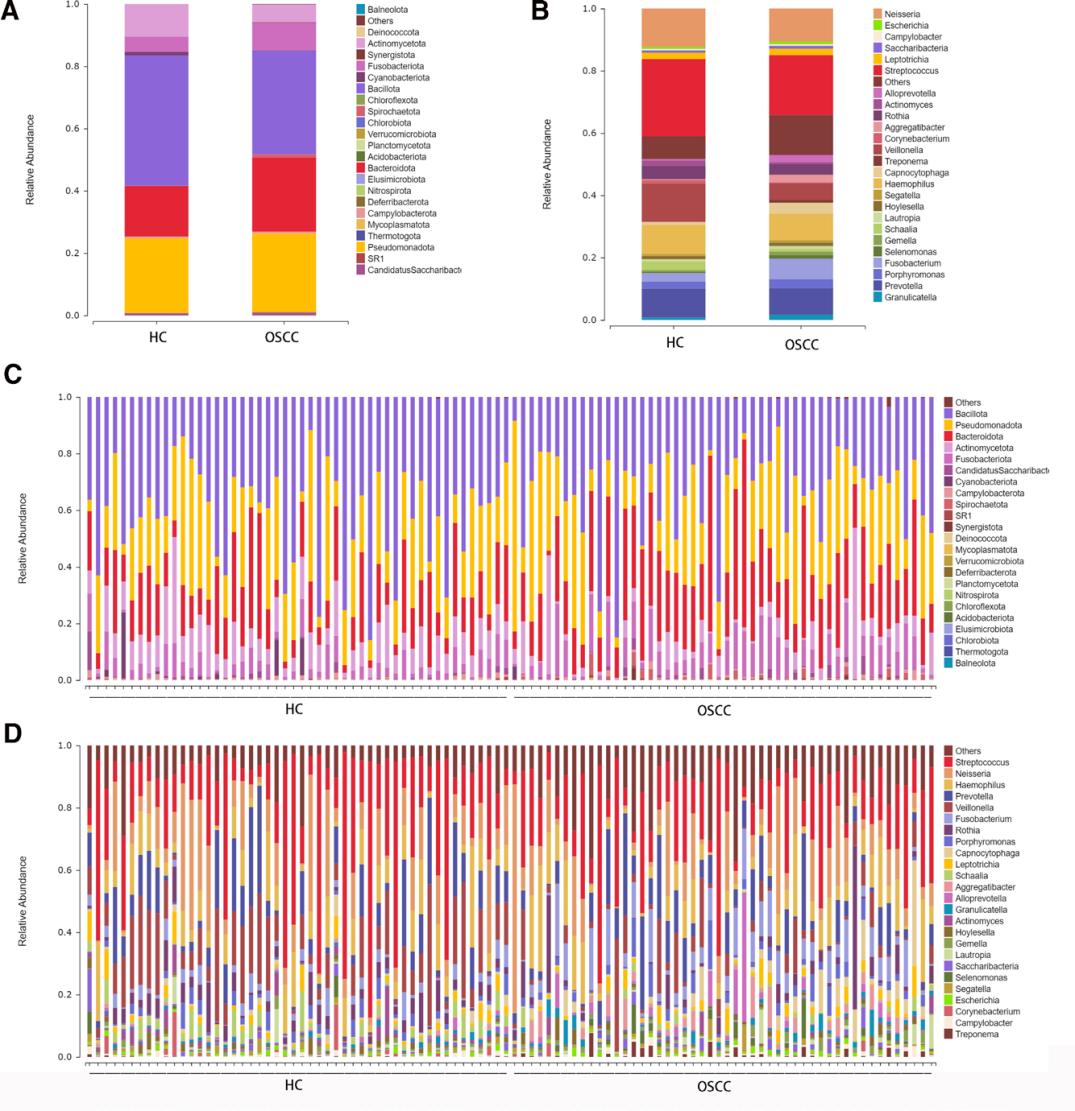
Taxonomy and relative abundance of bacterial communities in OSCC patients and healthy controls. (A) Phylum-level taxonomic composition and relative abundance of oral microbiota in the OSCC group and HC group. (B) Genus-level taxonomic composition and relative abundance of oral microbiota in OSCC and HC groups. (C) Phylum-level classification and relative abundance of bacterial communities in individual samples from OSCC and HC groups. (D) Genus-level classification and relative abundance of bacterial communities in individual samples from OSCC and HC groups. HC = healthy controls, OSCC = oral squamous cell carcinoma.

The above results confirmed that there were statistically significant differences in oral flora structure between OSCC group and HC group. In order to further analyze the relative abundance difference of oral salivary flora between OSCC group and HC group, the measured data were sequentially analyzed. Data showed that the top 5 oral bacteria groups in OSCC group were 41.86% Bacillota, 24.01%Pseudomonadota, 16.25% Bacteroidota, 10.35% Fusobacteriot and 4.92% Actinomycetota. In the HC group, the top 5 main components of oral salivarium flora were 33.31% Bacillota, 25.22% Pseudomonadota, 23.85% Bacteroidota, 8.85% Actinomycetota and 5.69% The composition of Fusobacteriot is shown in Table 3. The relative abundance of Bacillota and Fusobacteriot in OSCC group was lower than that in HC group, while the relative abundance of bacteroidota, pseudomonadota and actinomycetota in OSCC group was higher than that in HC group (Table 5, Fig. 5).

**Table 5:** Log10 conversion table of OSCC group and HC group relative abundance classification.

Name	HC (%)	Name	OSCC (%)
*Bacillota*	0.4186	*Bacillota*	0.3331
*Pseudomonadota*	0.2401	*Pseudomonadota*	0.2522
*Bacteroidota*	0.1625	*Bacteroidota*	0.2385
*Actinomycetota*	0.1035	*Fusobacteriota*	0.0885
*Fusobacteriota*	0.0492	*Actinomycetota*	0.0569

HC = healthy controls, OSCC = oral squamous cell carcinoma.

The relative abundance of oral flora in OSCC group and HC group was further explored at the genus level. The bacteria with high proportion in HC group were 24.87% Streptococcus, 12.32% Veillonella, 9.35% Haemophilus, 9.32% Prevotella, 4.33% Rothia and 2.78% Schaalia, 2.71% Fusobacterium, 2.22% Porphyromonas, 2.04%Leptotrichia, 1.69% Actinomyces, In contrast, the bacteria in OSCC group accounted for a higher proportion of 19.25% Streptococcus, 10.65%Neisseria, 8.52%Haemophilus, 8.50%Prevotella, 5.37%Veillonella, 3.54%Rothia, 3.48% Capnocytophaga, 2.89%Porphyromonas, 2.45%Aggregatibacter, 2.18%Alloprevotella are shown in Table 6

**Table 6:** Relative abundances of species in OSCC group and HC group.

Name	HC (%)	Name	OSCC (%)
*Streptococcus*	0.2487	*Streptococcus*	0.1925
*Veillonella*	0.1232	*Neisseria*	0.1065
*Haemophilus*	0.0935	*Haemophilus*	0.0852
*Prevotella*	0.0932	*Prevotella*	0.0850
*Rothia*	0.0433	*Veillonella*	0.0537
*Schaalia*	0.0278	*Rothia*	0.0354
*Fusobacterium*	0.0271	*Capnocytophaga*	0.0348
*Porphyromonas*	0.0222	*Porphyromonas*	0.0289
*Leptotrichia*	0.0204	*Aggregatibacter*	0.0245
*Actinomyces*	0.0169	*Alloprevotella*	0.0218

HC = healthy controls, OSCC = oral squamous cell carcinoma.

### 3.7. Analysis of species abundance difference between OSCC group and HC group

**Figure 6.**
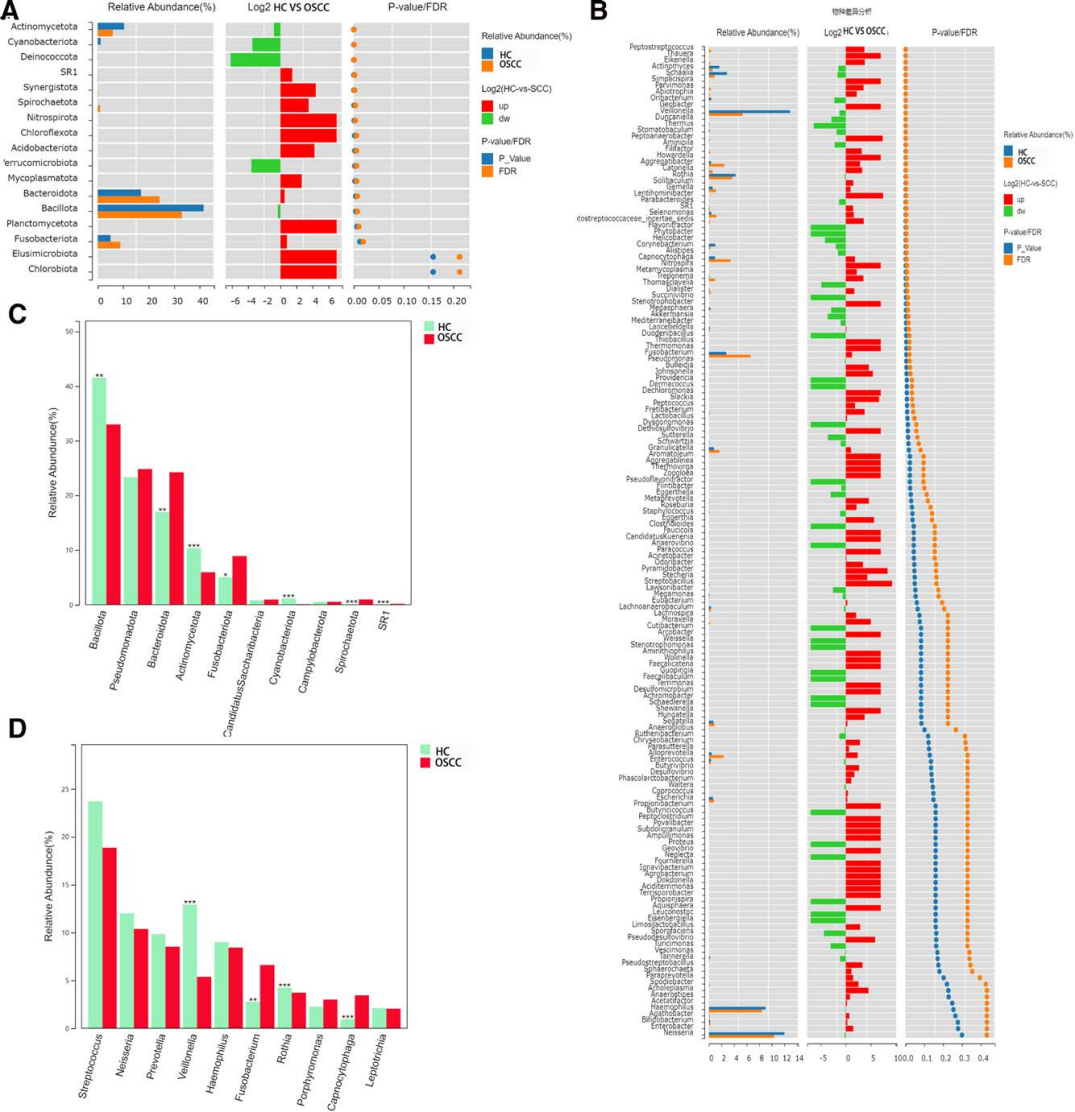
Relative abundance and differential analysis of microbial taxa between OSCC and healthy control (HC) groups. (A) Relative abundance of microbial phyla in OSCC and HC groups. The log2 fold changes (OSCC vs HC) of the phyla and corresponding *P*-values/FDR are also shown. Bacillota and Actinomycetota were significantly enriched in the HC group, while Bacteroidota and Fusobacteriot were more abundant in the OSCC group (*P <.05, **P <.01, ***P <.001). (B) Relative abundance of microbial genera in OSCC and HC groups with corresponding log2 fold changes and *P*-values/FDR. Significant differences were observed between several genera, including Fusobacterium and Veillonella, which were enriched in the OSCC group, while Rothia was more abundant in the HC group. (C) Phylum-level comparison of relative abundance between OSCC and HC groups, highlighting significant differences in several phyla (*P <.05, **P <.01, ***P <.001). (D) Genus-level comparison of relative abundance between OSCC and HC groups. Significant differences were found in genera such as Fusobacterium, Veillonella, and Rothia (***P <.001). FDR = false discovery rate, HC = healthy controls, OSCC = oral squamous cell carcinoma.

At the phylum level, the experiment found that Bacillota and Bacteroidota accounted for a relatively high proportion of microbial flora structure, and Bacillota accounted for 41.54% in HC group, which was significantly higher than OSCC group, and the difference was statistically significant (*P* <.01). The proportion of Bacteroidota in OSCC group was 24.21% higher than that in HC group, and the difference between the 2 groups was statistically significant (*P* <.01). Actinomycetota and Fusobacteriot also had a relatively high proportion in both groups. In the HC group, Actinomycetota accounted for 10.34%, respectively, and the HC group accounted for a higher proportion than the OSCC group. The difference between the 2 groups was statistically significant (*P *<.0001). In OSCC group, Fusobacteriot accounts for 8.89%, which is significantly higher than that in HC group, and the difference between the 2 groups is statistically significant (*P* <.05) (Fig. 6).

At the genus level, the data analysis results of OSCC group showed that Haemophilus, Fusobacterium and Veillonella were the dominant bacteria in 8.42%, 6.61% and 5.37%, respectively, and their corresponding proportions in HC group were 9.00%, 2.75% and 12.93%, respectively. The difference between the 2 groups was statistically significant in Fusobacterium and Veillonella (*P* <.001), but not in Haemophilus between the 2 groups.

In order to further explore the differences between the OSCC group and the HC group in the dominant species, species with the top 10 abundance were selected for difference analysis to show the average relative abundance of each group and the significance of the difference test. Bacillota, Bacteroidota (*P* <.001), Actinomycetota, Cyanobacteriota, Spirochaetota, SR1 (*P* <.0001) and Fusobacteriota (*P* <.05) showed the significant difference between the 2 groups at the phylum level. While Pseudomonadota, Candidatus, Saccharibacteria, Campylobacterota have no statistically significant difference between the 2 groups, the experimental results as shown in the Figure 6C. At the genus level, the difference between the 2 groups was analyzed, and the results showed that there was significant difference between the Veillonella, Rothia and Capnocytophaga groups (*P* <.0001). Fusobacterium had a statistically significant difference between the 2 groups (*P* <.001). However, there was no significant difference between Streptococcus, Neisseria, Prevotella, Haemophilus, Porphyromonas and Leptotrichia. Figure 6D for details.

### 3.8. Correlation analysis between oral microflora and host immunity

**Figure 7.**
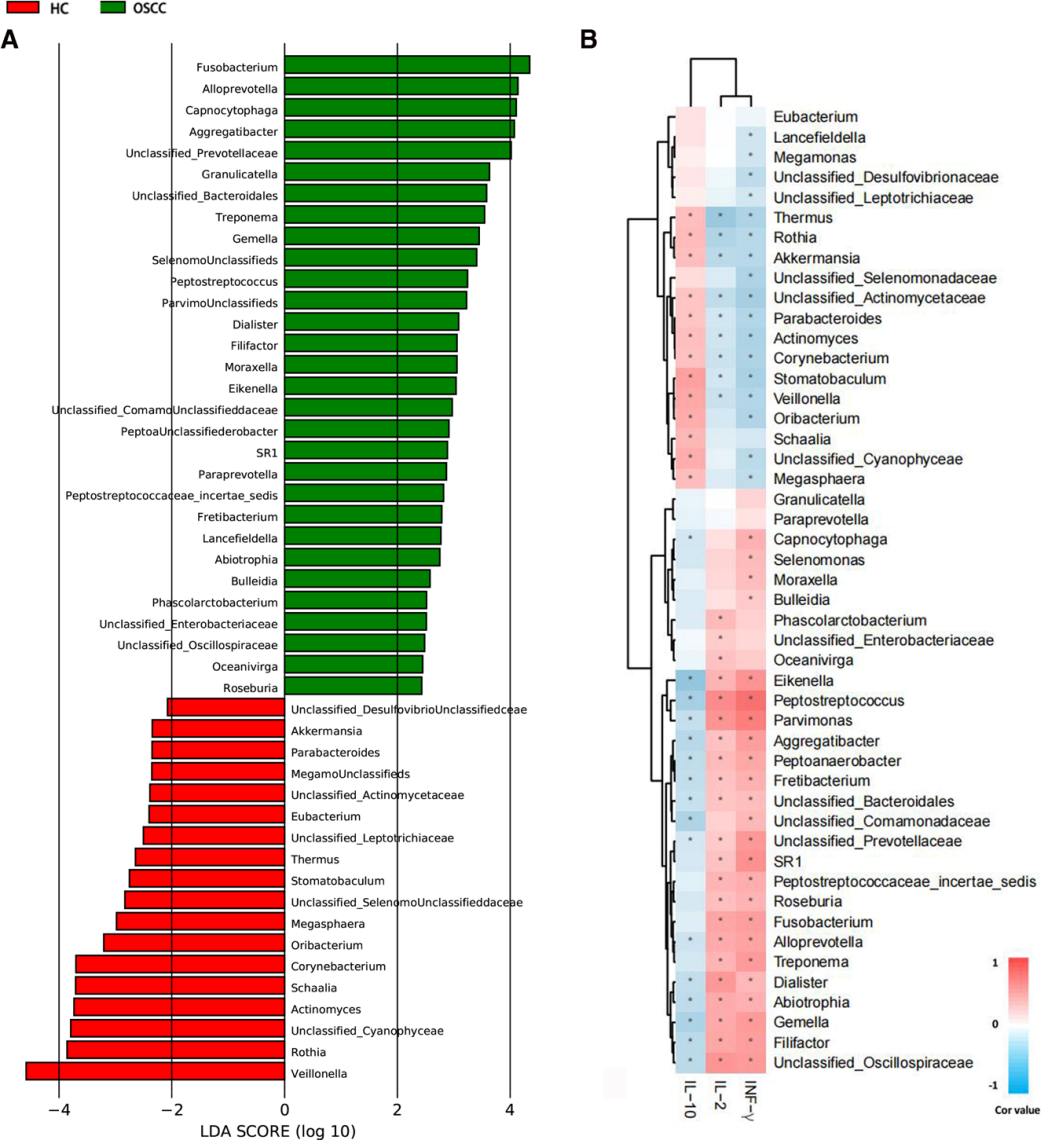
Differential abundance and correlation of bacterial genera with immune indices in OSCC and HC groups. (A) LDA effect size (LEfSe) results showing differentially abundant bacterial genera between OSCC and HC groups. Genera shown in green are more abundant in OSCC, while those in red are more abundant in HC, with LDA scores indicating the effect size of the difference (log scale). (B) Heatmap of Spearman correlation coefficients between differentially abundant genera and immune indices in OSCC and HC groups. The color intensity in each cell represents the correlation strength, with red indicating positive correlation and blue indicating negative correlation. Significant correlations (*P* <.05) are highlighted with an asterisk (*). HC = healthy controls, LDA = linear discriminant analysis, LEfSe = LDA effect size, OSCC = oral squamous cell carcinoma.

The results showed that through the difference analysis of microecological flora structure between the 2 groups, 48 bacteria genera with abundance difference between the 2 groups were finally screened (Fig. 7). In OSCC group, Fusobacterium, Alloprevotella, Capnocytophaga, Aggregatibacter and Unclassified_Prevotellaceae were found which showed a significantly increased trend compared with HC group. Veillonella, Rothia, Unclassified_Cyanophyceae, Actinomyces and Schaalia were significantly higher in HC group than in OSCC group A lot. See Figure 7A for details.

Correlation analysis was conducted between the above screened different bacteria genera and the concentration of immune indexes, and the Cor value between them was calculated by spearman correlation coefficient. In this experiment, the first 3 bacteria genera with the highest correlation with immune indexes and the absolute value of Cor ≥0.2 were selected, and the data with *P* <.05 were screened. Among the 48 differentially abundant bacterial genera identified, several demonstrated statistically significant correlations with systemic immune markers based on Spearman analysis (|*r*| ≥0.2, *P* <.05). Peptostreptococcus showed a strong positive correlation with IL-2 (*R* = 0.42, *P* = .003) and IFN-γ (*R* = 0.39, *P* = .005), and a negative correlation with IL-10 (*r* = −0.33, *P* = .012). Oralobacter was positively correlated with IL-10 (*R* = 0.36, *P* = .007), whereas Eikenella was negatively correlated with IL-10 (*r* = −0.31, *P* = .017). These associations suggest that specific salivary microbial taxa are linked to the systemic immune profile of OSCC patients.

## 4. Discussion

The human body harbors a tremendous multitude of microorganisms, encompassing a diverse array of species, with bacteria constituting the predominant majority. Experimental figures indicate that the population of resident bacteria in the human digestive system can reach 1014, and there is a wide variety of species, potentially numbering in the thousands.^[24]^ Out of them, approximately 54% have been subjected to cultivation and assigned names, 14% have been cultivated but not given names, and 32% are types of species that have not been cultured.^[25]^ Currently, our comprehension of the oral microbiota remains uncertain, and additional research is required to gather more empirical evidence to enhance and delve deeper into the understanding of the oral microflora.^[26]^

The bacteria that inhabit the oral microenvironment have a significant influence on both oral health and the development of systemic disorders. Research has demonstrated that oral microflora can create biofilms by co-aggregating on the surface of the mouth. The bacteria that colonize these biofilms preserve the integrity of their structure through interaction, while also ensuring that the host is not negatively impacted by the microbial flora.^[27]^ However, when the external environment changes, certain pathogenic bacteria that depend on specific conditions may undergo changes. This is evident in the increased growth of pathogenic bacteria and the disruption of the stable internal structure of the biofilm. As a result, the microecology of oral flora becomes imbalanced, posing a threat to the host’s health.^[28]^ In recent years, researchers have recognized the need to study and verify the disease-causing abilities of all bacteria in the mouth in order to gain a deeper understanding of oral health and disease.^[29]^ Studying the pathogenic potential of all bacteria is challenging due to the large number of oral bacteria and the symbiotic relationship between the normal flora in different parts of the human body and the host. This natural selection microecosystem complicates research and exploration of oral microflora.^[30]^

The identification and cultivation of bacteria in investigations of oral microbiota structure are commonly conducted using standard culture methods and independent molecular techniques, such as amplicon sequencing analysis targeting the 16S rRNA gene. For this trial, we collected saliva samples from individuals in 2 groups: the OSCC group and the HC group. Following amplicon sequencing analysis of the 16S rRNA gene, we observed distinct variations in the composition of oral microbiota between the 2 groups. When we analyzed the bacterial makeup in the 2 groups at the genus level, we found that in the OSCC group, the most common bacterial groups were Haemophilus, Clostridium, and Veyonia, making up 8.42%, 6.61%, and 5.37% of the total, respectively. Multiple studies have demonstrated that Haemophilus is considerably present in chronic obstructive pulmonary disease, lung cancer, and head and neck cancer.^[31,32]^ Similar to other bacterial infections, Haemophilus may induce inflammation and trigger the release of numerous cytokines and chemokines. It also activates the NF-κB pathway in epithelial cells, leading to a notable increase in the expression and release of pro-inflammatory mediators such as IL-6 and IL-8. These processes have an impact on human health.^[7,33]^ Veillonella is a prevalent microorganism that inhabits several parts of the human body, such as the mouth cavity, gastrointestinal tract, and vagina. When the microecological structure of it is disrupted and altered, it can also function as a pathogen causing infections in the sinus, lung, heart, bone, and central nervous system.^[34,35]^ In the present study, we conducted a statistical comparison of the salivary microbial composition between OSCC patients and HC. A total of 48 genera with significantly different abundance profiles were identified. Correlation analyses revealed significant associations between several of these genera and systemic immune parameters. Notably, Peptostreptococcus demonstrated a strong positive correlation with IL-2 (*R* = 0.42, *P* = .003) and IFN-γ (*R* = 0.39, *P* = .005), and a significant negative correlation with IL-10 (*r* = −0.33, *P* = .012). These findings suggest that Peptostreptococcus may contribute to immunological modulation in the tumor microenvironment, potentially influencing OSCC progression through its impact on pro- and anti-inflammatory cytokine expression. According to reports, it is believed that [insert specific microbe] plays a vital role in the development of oral disorders and digestive tract diseases. Its mode of action is thought to involve initially colonizing the mouth and then adhering to the surface of precursor microorganisms.^[36,37]^ Alteration of the environment occurs by means of the synthesis and release of metabolites, which frequently stimulate the proliferation of other organisms.^[36]^ The process of microbial succession and the subsequent rise in variety will result in an imbalance of the microflora structure. This, in turn, will alter the structural, metabolic, and chemical interactions among bacteria, ultimately impacting the health of the organism.^[38,39]^

To further interpret our findings, it is essential to consider the immunomodulatory roles of specific bacterial taxa identified in this study. The salivary microbiota of OSCC patients exhibited notable alterations compared to HC, with increased abundance of genera such as Fusobacterium, Capnocytophaga, and Peptostreptococcus, and a reduction in Veillonella and Rothia. These microbial shifts were significantly correlated with variations in systemic cytokine levels, including IL-2, IL-10, and IFN-γ. Peptostreptococcus, for example, was positively associated with IL-2 and IFN-γ, and negatively with IL-10. This suggests a skewing toward a Th1-type immune response, characterized by enhanced cellular immunity and pro-inflammatory activity. IL-2 and IFN-γ are critical cytokines involved in the activation of cytotoxic T lymphocytes and natural killer (NK) cells, which play key roles in antitumor immunity. However, chronic activation of these pathways may also contribute to sustained inflammation, tissue damage, and immune escape mechanisms within the tumor microenvironment.^[40,41]^ Conversely, IL-10, typically regarded as an anti-inflammatory cytokine, was inversely associated with the abundance of Peptostreptococcus and Eikenella. A reduced IL-10 level may impair immune regulation and promote a pro-inflammatory milieu that facilitates tumor progression. Additionally, Fusobacterium, previously implicated in colorectal and head and neck cancers, is known to induce the expression of immune checkpoint molecules and modulate tumor-infiltrating lymphocytes, thereby supporting immune evasion.^[42]^ The observed correlations suggest that specific oral microbial species may not only reflect the immune status of the host but also actively participate in reshaping the tumor-associated immune landscape. Microbial-derived products, such as lipopolysaccharides, short-chain fatty acids, and other metabolites, can trigger pattern recognition receptors (e.g., TLRs) on immune and epithelial cells, leading to downstream cytokine release and modulation of immune cell recruitment and activation. These host–microbiome interactions may establish a permissive environment for cancer initiation and progression.^[43]^ Overall, our results highlight the potential bidirectional relationship between salivary microbiota dysbiosis and host immune responses in OSCC. The presence of specific bacterial genera may influence tumor-related immunity through modulation of cytokine production, and in turn, the tumor immune microenvironment may shape microbial community composition. Further studies are warranted to elucidate the causal relationships and underlying mechanisms.

## 5. Conclusion

This study demonstrated significant differences in both immune factor expression and oral microbial diversity between OSCC patients and HC. Elevated levels of IL-2 and IFN-γ, and decreased IL-10 were observed in OSCC patients, correlating with tumor differentiation and stage. Moreover, distinct bacterial taxa, such as Fusobacterium and Veillonella, were associated with immune responses, indicating potential interactions between oral microbiota and systemic immunity in OSCC. These findings suggest that microbial dysbiosis may contribute to OSCC progression through modulation of the host immune microenvironment.

## Acknowledgments

We sincerely thank everyone who participated in this research.

## Author contributions

**Conceptualization:** Jiazhi Xu, Jun Zhao.

**Data curation:** Jiazhi Xu, Jun Zhao, Rui Bai, Jiayi Hang.

**Formal analysis:** Jiazhi Xu, Jun Zhao, Chan Tang, Jiayi Hang.

**Investigation:** Jiazhi Xu, Dongxiao Nong, Jun Zhao, Rui Bai.

**Methodology:** Jiazhi Xu, Dongxiao Nong, Xiaolin Nong, Jun Zhao, Rui Bai, Chan Tang, Jiayi Hang.

**Resources:** Jiazhi Xu, Xiaolin Nong, Rui Bai, Chan Tang, Jiayi Hang.

**Software:** Chan Tang.

**Writing – original draft:** Jiazhi Xu, Dongxiao Nong, Xiaolin Nong.

**Writing – review & editing:** Xiaolin Nong.
